# The object space task shows cumulative memory expression in both mice and rats

**DOI:** 10.1371/journal.pbio.3000322

**Published:** 2019-06-17

**Authors:** Lisa Genzel, Evelien Schut, Tim Schröder, Ronny Eichler, Mehdi Khamassi, Angela Gomez, Irene Navarro Lobato, Francesco Battaglia

**Affiliations:** 1 Donders Institute for Brain Cognition and Behaviour, Radboud University, Nijmegen, the Netherlands; 2 RadboudUMC, Nijmegen, the Netherlands; 3 Institute of Intelligent Systems and Robotics, Sorbonne Université, CNRS, Paris, France; Institute of Science and Technology Austria, AUSTRIA

## Abstract

Declarative memory encompasses representations of specific events as well as knowledge extracted by accumulation over multiple episodes. To investigate how these different sorts of memories are created, we developed a new behavioral task in rodents. The task consists of 3 distinct conditions (stable, overlapping, and random). Rodents are exposed to multiple sample trials, in which they explore objects in specific spatial arrangements, with object identity changing from trial to trial. In the stable condition, the locations are constant during all sample trials even though the objects themselves change; in the test trial, 1 object’s location is changed. In the random condition, object locations are presented in the sample phase without a specific spatial pattern. In the overlapping condition, 1 location is shared (overlapping) between all trials, while the other location changes during sample trials. We show that in the overlapping condition, instead of only remembering the last sample trial, rodents form a cumulative memory of the sample trials. Here, we could show that both mice and rats can accumulate information across multiple trials and express a long-term abstracted memory.

## Abstract 2

Memories are stored and retrieved in different ways, depending on the age of memory and the character of the information, but many tasks for rodent subjects cannot differentiate between them. A new training paradigm for rats and mice enables testing for abstracted memory representation while controlling for memory recency.

## Introduction

Memories are stored and retrieved differently depending on the age of memory and the character of memorized information. In episodic memory, the details of the memorized event are retained. Conversely, semantic memory extracts general, statistical knowledge across multiple events. Memory consolidation processes may promote a transition between these 2 types of memory organization [1,2]. However, many tasks, especially for rodent subjects, cannot differentiate between the two, even though this differentiation is critical to further our understanding of memory mechanism [3]. While much effort has been made into developing rodent tasks testing aspects of episodic memory (what, where, when) [4,5], few studies attempt to test semantic-like memory while controlling for individual event memories. Here, we address this gap with the Object Space Task.

Most memory paradigms used in rodents can be trained in a short time (1–2 sessions), enabling one to determine exact timings of memory interventions. But many such protocols require aversive reinforcers, such as electrical shocks in fear conditioning or avoidance learning [6,7] or other strong motivators such as water or food reward. Such learning incentives strongly drive the neuromodulatory systems, a fact often not considered in studies using these tasks [8]. In contrast, object recognition paradigms make use of a rodent’s natural tendency to explore more novel items, thus allowing for the investigation of memory processes without an intrinsic, difficult-to-control side effect on motivation and emotion [9–11].

Another critical factor influencing memory acquisition is the frequency of events the animal experiences. In some memory tasks, the animal is exposed to repeated training trials. Spatial memory tasks such as the water maze consist of multiple sample trials for the rodent to learn the location of a hidden platform, most commonly trained across multiple days [12]. The radial arm maze requires animals to repeatedly sample baited arms, and their memory performance is assessed by the number of errors, namely the frequency of unbaited arm visits within a given trial; again, days to weeks of training are needed for the animal to perform above chance level [13,14]. Similarly, in some aversive conditioning paradigms, subjects undergo multiple pairings of a conditioned stimulus (CS), such as a tone or light with a mild foot shock [7]. In other memory tasks, the animal only experiences a single event, which is the case in some fear memory paradigms, object recognition, or object displacement memory [15–17]. In object tasks, animals are allowed to explore 2 objects in a given environment for a certain amount of time. After a delay—a short delay to assess short-term memory or a delay of 24 hrs to assess long-term memory—one of the objects is either replaced by a novel object (testing object recognition memory) or moved to a novel location (testing object position memory). Memory is assessed by calculating the difference in exploration time of the (for rodents preferred) novel item/location versus the familiar. In tasks for which the number of events the animal experiences varies greatly, it is unclear which part of the training was significant to the animal’s performance. Is only the most recent event memorized by the animal? Or can memory be accumulated across extensive time periods or multiple trials?

These questions are key to understanding mechanisms of episodic versus semantic memory [3], but they are difficult to address in most memory tasks. However, some recent work has attempted to study the accumulation of evidence across multiple events. An example is a modified version of the water maze, in which evidence accumulation was assessed as mice were trained on multiple platform locations that were drawn stochastically from a specific spatial distribution, and retrieval of “averaged” memory of the learned platform locations was assessed after a 1-day or 30-day delay [18]. Another example is paired-associate learning in rodents, in which memory of flavor–place associations is gradually learned with repeated trials over weeks, but later, updating can occur within 1 trial [19–21].

Training procedures in these cumulative memory tasks are often lengthy and labor intensive. In addition, information can be mainly acquired from either retrieval and/or updating; encoding and consolidation processes are difficult to study. A water-based paradigm, such as that of Richards and colleagues [18], is ill-suited for electrophysiological recording of brain activity during learning. We overcame these limitations by developing a task designed to extract information from multiple similar events accompanied by suitable control conditions. The task is a variation on the traditional object–place memory task, exploiting a rodent’s natural tendency to explore novel configurations. In the object space task, we manipulate the stability of different components of the experience (here, specifically, object position), making them more or less amenable to accumulation across episodes. We developed task versions that are suitable for both rats and mice.

In this new task, rodents are allowed to explore 2 objects presented across multiple trials in a stable, overlapping, or random sequence. In all conditions, the actual objects change from trial to trial so that the emphasis is on the spatial configurations. In the stable condition, objects are always presented in the same location across sample trials (see Figs 2 and 3). In the test trial, after a delay, 1 object is moved to a novel location. We expect to see a preference for the object in the novel location in the test trial but no preference for either location over the course of training. This condition can be solved by remembering only the final sample trial (that is, using episodic-like memory or familiarity) or by creating a cumulative memory of all sample trials. The overlapping condition is our key condition. One object location remains stable across sample trials whereas the other object moves between 1 of 3 locations each sample trial. Importantly, the last sample trial shows the same configuration as the test trial after a delay (but with different objects). Thus, if the animal only remembers the most recent event it experienced, it will show no preference for either object location since both locations are familiar to the animal. Conversely, if the animal has accumulated the overlapping information of the stable location over time, it will still show a preference for the location less often shown. The control random condition consists of objects presented in random spatial configurations (with each location appearing the same amount across trials) in which no patterns can be extracted and no place preference should develop.

The 3 conditions can be repeated multiple times in the same animals, thereby allowing for within-subject designs controlling for baseline differences between animals. Object identity changes from trial to trial in order to keep exploration duration constant. Further, it is easy to combine behavioral training with physiological measures, such as electrophysiology and other manipulations. The rat version of this task requires only 1 day of training, involving 5 trials with 50-min intertrial intervals and a 10-min test trial follows 24 hrs later. In mice, this training protocol is repeated over the course of 4 days, and a test trial follows 24 hrs after the fourth training day. We further ran a 4-week training paradigm in mice and could show that cumulative memory was retained for at least 5 days. We show here that both rats and mice can express a cumulative memory.

## Methods

### Ethics statement

In compliance with Dutch law and institutional regulations, all animal procedures were approved by the Central Commissie Dierproeven (CCD) and conducted in accordance with the Experiments on Animals Act (project number 2016–014 and protocol number 001 and 005).

### Subjects

Male C57Bl6/J mice 7–8 weeks of age at the start of behavioral training (Charles River) and male Lister Hooded rats (12 weeks, Charles River) were group housed with ad libitum access to food and water. Animals were maintained on a 12-hr light/dark cycle and tested during the light period.

### Behavioral training

#### Habituation

Animals were thoroughly handled in their second week after arrival in the animal facility. Each animal was actively handled daily for at least 5 min. We emphasize here that handling of the animals is extremely important. Picking them up by the tail is aversive, and inadequate handling can affect the animal’s performance on multiple tasks [22]. Mice and rats were handled so that they typically climbed by themselves on the experimenter’s hands when taking them out of the home cage and out of the training arena (see handling video at https://www.memorydynamics.org/#/animal-handling/). After handling, animals were habituated to a square arena (75 cm x 75 cm) for 5 sessions within 5 days (can also be performed in 3 days; see methods for implanted animals below). Some handling can be combined with habituation; however, a minimum of handling is needed beforehand for the habituation itself to be less fearful. The walls and the floor were white or green to facilitate background subtraction in video analysis software. On the bottom side of the floor, magnets were placed in 4 locations for easy and consistent placement of the objects; objects were affixed to square metal plates (S1 Fig). In the first habituation session, the animals were allowed to explore the box together with all cage mates for 30 min. In the second and third session, they were placed in the box individually for 10 min. In the final 2 sessions of habituation, 2 objects (towers made from Duplo blocks, not used in main experiment) were placed in the box at locations not used during training, and the animals were allowed to explore for 10 min.

#### Training

The object space task consists of 3 conditions: stable, overlapping, and random, as described above (see Figs 2 and 3). Conditions, sequences, and locations (identity of “stable” and “less stable”) were counterbalanced among animals and sessions, and the experimenter was blinded to the condition (see Data acquisition below). All locations in all conditions (i.e., which was the “nonmoving” location in stable and overlapping) were counterbalanced across animals within each condition. Further, within animals, different locations were used for stable and overlapping to avoid the training of general location preferences and interferences from one condition to the next. Also, the “moving” locations were counterbalanced across animals within each condition. In random, locations were chosen pseudo-random, with all locations using the same amount of times per day, and each location was used at least once in 3 and the last 2 trials of the day, respectively. Finally, the sequence of the conditions (e.g., first stable, then random, last overlapping) was also counterbalanced across animals.

At the beginning of each session (2 days for rats, 5 days for mice), cues were placed on the walls inside the box, distributed intentionally nonsymmetric, and kept constant across all trials within 1 session. Thus, cues were typically not placed in the middle of each wall but would rather be distributed in a way that one cue would, for example, cover the lower left part of the wall while another cue would occupy the top right part of another wall. At least one 3D cue was placed above any of the walls to facilitate allocentric processing during the task. All cues were chosen to be high contrast and varied from session to session in general shape and geometry to cater to the poor vision of rodents. A camera was placed above the box to record every trial and to allow for online scoring of exploration time. Behavior was manually scored as exploration when the animal approached and then touched/sniffed/climbed/sat upon the object. Extensive chewing of the object or grooming/sleeping while on the object would have not counted as exploration but also did not occur here. Multiple experimenters were involved in the experiment, and each separate batch of animals (*n* = 8 per batch) was trained by either 1 constant experimenter or by at least 2 experimenters in a rotational schedule; these differences had no effect on the replicability of the results from one batch of animals to the next.

In each condition, animals were allowed to explore 2 objects for 5 min, with an intertrial interval of 30 min for mice, 50 min for rats. Mice were trained interleaved in groups of 4 with 2 groups per day (morning/afternoon), rats in groups of 8 (1 group per day). Smaller group sizes for interleaving and thus shorter intertrial intervals (30 min versus 50 min) were chosen for mice due to their decreased ability of forming rapid memories in contrast to rats. The tighter reinforcement schedule (shorter ITIs) should thus lead to equal memory strength. Before the beginning of each sample trial, the box and the objects were thoroughly cleaned with 70% ethanol. Each sample trial consisted of a different pair of matching objects varying in height, width, texture, and material (including metal, glass, hard plastic, and lacquered wood; see S1 Fig for example objects) to keep exploration times constant across trials and to focus the memory task on spatial configurations and not object identity. Object sizes ranged from 4–26 cm in height to 5–18 cm in width. Objects were glued onto metal coasters and placed onto the magnets that were fixed on the floor of the arena.

Objects were never repeated during the training period of 1 condition (1 session). Rats received 5 sample trials in total. This procedure was repeated in mice over the course of 4 consecutive days in which they were presented with either 3 sample trials per day (see S2 Fig) or 5 sample trials per day, thus accumulating 12 or 20 total sample trials. The test trial, 24 hrs after the last sample trial, consisted of again 2 objects, and animals were allowed to explore for 10 min; however, only the initial 5 min were used (for 10-min results, see S3 Fig).

In each species, 4 batches of each 8 animals were run, resulting in a total of 32 animals. In mice, 1 animal was excluded after running the first experiment (3-trial version) due to exploration times of less than 5 sec and never experienced the 5-trial version. In rats, 1 animal was excluded due to exploration times of less than 5 seconds, and the data of another animal was not included due to false placement of the objects during the test in the overlapping condition (not included in the other conditions due to the within-subject analysis).

Additionally, 8 mice were run on a 4 week version of the overlapping condition, with 3 weeks of each 25 trials (5 days, Mo–Fri) and a final trial on Wednesday of week 4. The second week’s Monday (trial 26) as well as the final trial (trial 76) were run with the same configuration as the previous sample trial to function as 3-day and 5-day test trials. All animals were included in the analysis.

#### Data acquisition

**Fig 1:**
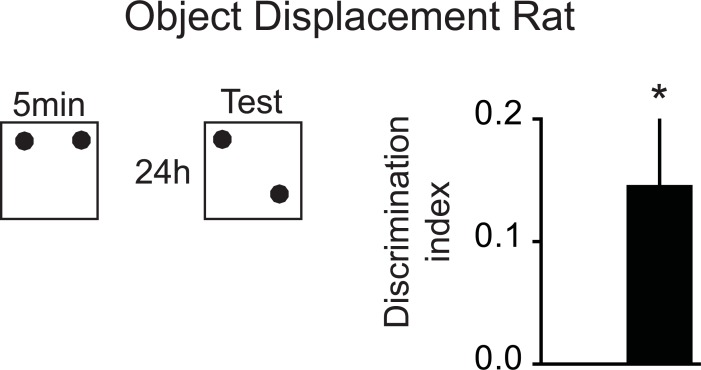
Object displacement rat. For comparison, DI for a classic version of the object displacement task with 5-min sample and 24 hr test (same object both trials). *n* = 8, *P < 0.05 to chance. Data in S5 Data. DI, discrimination index.

We developed an in-house program for training and scoring. The Object Scorer reads previously prepared training sheets with the object and locations for each trial of each animal, presents this information at the beginning of each trial to the experimenter (see Fig 1), and automatically extracts exploration times from the manually scored videos. Therefore, the operator cannot keep track of which animal is in which condition and which is the stable versus moved object for each trial, and he or she can be considered blind. Source code for the Object Scorer software is available at https://github.com/MemDynLab/Score.

#### Statistical analysis

The discrimination index (DI) used to assess memory performance was calculated as the difference in time exploring the novel object location and stable location divided by the total exploration time. This results in a score ranging from −1 (preference for the stable location) to +1 (preference for the moving object location). A score of 0 indicates no preference for either object location. Total exploration time and DI were assessed with repeated-measure ANOVAs with factors condition and trial (6 trials) in rats. Additionally, in rats, the DI of the test trial was assessed with a repeated-measure ANOVA for condition. Due to the different training schedule in mice (5 trials per day for 4 days but only 1 test trial on day 5), the sample trials were separately assessed by including the factors condition, trial, and day across the 20 sample trials in mice in an repeated-measure ANOVA. To test long-term memory in mice, the final sample and test trial were included in a repeated-measure ANOVA with factors condition and trial. When a significant main effect or interaction was found, one-sample *t* tests were performed to analyze memory performance with respect to chance level in the last sample trial and test trial.

The 4-week overlapping training was analyzed with repeated-measure ANOVA for week (each animal averaged across the week), for exploration time, and for discrimiation index. Further, the 2 test trials (3 day and 5 day) were tested to chance level with a one-sample *t* test.

### Implanted animals

#### In vivo calcium imaging of neurons in the medial prefrontal cortex in mice

C57Bl6/J mice, 7–8 weeks of age (Charles River) at the time of surgery, were group housed with ad libitum access to food and water. Excitatory neurons were labeled by injecting an adeno-associated virus (AAV, serotype 2/5) driving expression of GCaMP6f under the control of the CaMKIIa promoter, targeting prelimbic area of the medial prefrontal cortex (AP: +2.0, ML: −0.4, DV: −2.5, −1.9). Two weeks after viral transfection, a microendoscope probe (GRINTECH) was implanted above the transfected tissue. After 3 weeks of recovery, the miniature microscope (miniscope, Inscopix, Inc) was lowered above the microendocscope until cells came into focus. Then, a baseplate was fixed to the skull to preserve appropriate focus and serve as a docking station for the miniature microscope. The next day, animals were extensively handled and habituated to a dummy miniscope for 2 consecutive days by allowing the mouse to roam freely around the home cage with the dummy miniscope attached for 5 min each day. Importantly, fixation of the animal or anesthesia prior to attaching the miniscope is unnecessary if the animal is well handled. Next, mice were habituated to the arena for the object space task according to the protocol previously described; however, the 5 sessions were performed across 3 days. During the individual habituation sessions, the real miniscope was attached to the animal’s baseplate to ensure proper habituation to the device before the start of behavioral training.

On the first day of training, the surface of the microendoscope was thoroughly cleaned with lens cleaner and lens paper. The miniscope was attached to the baseplate, and proper focus of the field of view was established by using the nVista acquisition software (Inscopix, Inc). The animal was then placed in an empty cage for 10 min before the start of training. This cage also provided as a resting cage during the intertrial intervals. Recordings were taken from each 5-min sample trial and during each intertrial interval for another 5 min. To reduce risk of photobleaching, the blue LED was switched off for at least 5 min in between recording sessions. After the animal had completed the training day, the protocol was repeated for the next animal.

#### In vivo electrophysiology in the medial prefrontal cortex and hippocampus in rats

Lister Hooded rats of 2 months of age (Charles River) were group housed with a 12-hr:12-hr light:dark cycle, and water and food were provided ad libitum. After surgery, animals were individually housed with environmental enrichment (wood blocks). Previous to the surgery, animals were handled and accommodated to eat “Weetos” cereals (Kellog’s) for 5 consecutive days. The last 3 days of handling, rats were habituated to the wooden sleep boxes (75 cm x 35 cm x 50 cm) containing bedding material for 2 hrs. In order to perform simultaneous chronic extracellular recording in CA1 of the hippocampus and prelimbic area of medial prefrontal cortex (mPFC), a modified light custom-built microdrive design containing 16 movable tetrodes was implanted on the animal’s head by stereotaxic surgery (6 in CA1 and 10 in mPFC). Under isofluorane anesthesia, two 3 mm AP x 2 mm ML and 1 mm x 1 mm craneotomies in the right hemisphere were made above the prelimbic area (AP: +3.5 mm, ML: −0.5 mm from bregma) and CA1 (AP: −3.8 mm, ML: −2 mm from bregma), respectively. One small screw (M1 x 3) was driven into the bone above the cerebellum as a ground electrode for recordings and other 4 additional screws were fixed to the skull to stabilize the structure. After carefully removing the dura mater, polyamide tubes bundles were placed on the cortical surface, and subsequently, the cortical surface was covered with sterile vaseline. Screws and microdive were cemented to the skull using quick adhesive cement (C&B Metabond) and dental acrylic cement. In the next days to implantation, animals were placed in the sleep box, signal was checked, and electrodes were gradually driven into the brain target area over the course of up to 1.5 weeks. After 1 week of recovery, rats were handled and habituated to the arena according to the protocol previously described but across 3 days. Once the target area was reached, the recording sessions started for all the conditions of object location task previously mentioned (stable, overlapping, and random). Using the Open Ephys recording system combined with Bonsai software, electrophysiological recording took place during all training and test trials in the arena, as well as during 45 min before training (presleep), 45 min intertrial intervals, and 3 hrs after training in the sleep box. The head stage plugging prior to recordings did not cause any stress on animals due to the well handling and “Weeto” cereals animal fondness.

### Computational modeling of memory traces

To characterize the build-up of a memory trace and its expression, we developed and simulated a computational model that progressively learns place–object associations and makes decisions about which proportion of time to spend exploring each object in order to minimize uncertainty about these place–object associations. The model employs 2 parameters: a learning rate *α*, which determines the balance between recent and remote memories, and a parameter *β*, which determines the balance between neophilic (preference for more novel object location) and neophobic (avertion for more novel object location) exploratory behaviors by changing the weight assigned by the model to “100% probability” locations (i.e., locations that are either occupied by an object or free with very high probability) to “uncertain” locations (e.g., a location that is occupied half of the time).

For each discrete object *o*_*k*_∈{*o*_1_,*o*_2_} and for each of the 4 possible locations *l*_*i*_∈{*l*_1_,*l*_2_,*l*_3_,*l*_4_}, the model maintains a memory trace *m*_*t*_(*o*_*k*_,*l*_*i*_), which counts how many times object *o*_*k*_ has been observed in location *l*_*i*_. However, instead of remembering a perfect count of place–object associations over time, the model rather progressively updates this memory trace at each trial *t* by integrating each new observation with a low-pass filter:

$$m_{t+1}(o,l)\leftarrow {(1-\alpha )m}_{t}(o,l)+\alpha \delta_{o,l}$$ (1)

with the initial memory trace at time *t* = 0 initialized to *m*_0_ = 0, and in which *δ*_*o*,*l*_ is a Kronecker delta so that

$$\delta_{o,l}=\left\{ \begin{array}{c} 1,if\;object\;o\;is\;observed\;in\;location\;l\\ 0,otherwise \end{array}\right.$$

*α* is a learning rate (0≤*α*≤1), which determines the balance between recent and remote memories. If *α* is high (i.e., close to 1), the model “forgets” the past memory of a given place–object association (*o*,*l*) at each new observation *δ*_*o*,*l*_ so that the updated memory trace *m*_*t*_(*o*,*l*) at the end of trial *t* only reflects information gathered during this last trial, producing a result comparable to what would be observed if behavior was guided by episodic memory of the last observation. Thus, a high *α* would predict bad performance in the test trial of the overlapping condition because only the last training trial would be remembered, for which there is no distinction between the moving object from the stable one and the past trials would be forgotten. Conversely, if *α* is low (i.e., close to 0), each new observation *δ*_*o*,*l*_ only updates to a small extent the memory trace, and many trials with object *o* at the same location *l* are needed to build a reliable memory of this place–object association. Thus, *α* determines the time scale over which information is retained and integrated.

The second component of the model will allocate proportions of exploration time to the 2 objects after observing their current locations. Such decision-making in the model is guided by the uncertainty in the location associated to each object. After normalizing the memory traces over all locations for a given object *o* (so that they add up to 1), we compute the entropy *S* in this distribution:

$$S_{t}(o)=-\sum_{i=1}^{4}m_{t}(o,l_{i})log(m_{t}(o,l_{i}))$$ (2)

The more uncertainty in the usual location associated to that object (if the object is always moved), the more exploring it and its present location might help gain information (and thus reduce uncertainty) about the object's actual location. Conversely, the less uncertainty in the location associated to that object (if the object is always at the same position), the less information can be acquired by exploring it again at the same location. Thus, rather than making decision based on extrinsic rewards (which are absent from this task), the model makes decisions driven by intrinsic motivations relative to information that can be acquired about regularities of its surrounding environment [23–25].

In practice, the proportion of time spent exploring each object *P*_*t*+1_(*o*_*k*_|*m*_*t*+1_) is computed from the uncertainty associated to the objects *S*_*t*_(*o*) based on a Boltzmann softmax equation:

$$P_{t+1}(o_{k}|m_{t+1})=\frac{exp(\beta S_{t}(o_{k}))}{\sum_{j\neq k}exp(\beta S_{t}(o_{j}))}$$ (3)

in which the second parameter of the model, *β*, both determines the sign of the effect (*β*>0 means neophilia, i.e., attraction by the object with highest uncertainty; *β*<0 means neophobia, i.e., attraction by the object with lowest uncertainty) and the magnitude of the effect (|*β*| close to 0 will lead to little preference for an object over the other; |*β*| close to ∞ will lead to very high preference for the uncertain object).

#### Model simulation

First, we verified that the model can reproduce the animals’ general behavioral tendencies [26]. We simulated the model on exactly the same series of place–object locations experienced by the animals during different sessions of the task. We explored different combinations of values for (*α*,*β*) in [0;1]×[−1;1], with steps of 0.1, and found that *α* = 0.6 and *β* = 0.2 were roughly reproducing the curves of the average behavior observed over the populations of rats and mice. In a second step of simulation, after finding the parameters that best account for each animal’s behavior (see model fitting procedure below), we simulated again the model with these parameters, then measured the DIs in stable, random, and overlapping conditions and performed the same statistical tests as for animal data to determine significant differences.

#### Model fitting

The aim here was to find for each individual subject, rat or mouse, the values of the model *M*’s parameter set *θ* (here *α* and *β*) that best fit the data by maximizing the log-likelihood *LL* of the trial-by-trial sequence of proportions of time spent exploring object *o*_1_ relative to object *o*_2_, as observed in the experimental dataset *D*:

$$\theta_{opt}=\arg\;\max_{\theta }\left\{log(P(D|M,\theta ))\right\}$$ (4)

$${LL}_{opt}=\max_{\theta }\left\{log(D|M,\theta )\right\}$$ (5)

We fitted the model separately on the data for each condition (stable, random, overlapping) in order to observe potential differences in the optimized parameters between conditions. In order to search for the model’s *LL*_*opt*_ and *θ*_*opt*_, we employed a gradient-descent method with Matlab’s *fminsearch* function initialized at 1,000 initial parameter-sets (*α*,*β*) randomly sampled in [0;1]×]−∞;∞[. We moreover verified that the model was fitting the data better than chance by computing a likelihood ratio test comparing *LL*_*opt*_ with the log-likelihood obtained with a model that always explores 50% object *o*_1_ and 50% object *o*_2_.

#### Model comparison

In order to verify that the present model (hereafter named *M*_1_) with 2 parameters (*α* and *β*) both provided the most accurate and parsimonious account of the data, we compared it with alternative models. These alternative models were extensions of model *M*_1_ with additional parameters; model *M*_2_ also included a forgetting parameter *γ* [27], which modulated the degradation of memory traces from 1 day to the next:

$$m_{t+1}(o,l)\leftarrow m_{t}(o,l)+(1-\gamma )(m_{0}-m_{t}(o,l))$$ (6)

in which 0≤*γ*≤1 and *m*_0_ = 0 is the initial value of all memory traces (also used for Eq 1 above) and which constitutes an attractor toward which the forgetting mechanism is pulling memory traces over time. Model *M*_3_ was an extension of model *M*_2_ in which *m*_0_ was a free parameter optimized on the data rather than being fixed. Finally, model *M*_4_ was an extension of model *M*_1_ in which *m*_0_ was also a free parameter but no forgetting mechanism was included.

We compared the ability of the different models to fit the data while at the same time penalizing for their complexity (i.e., penalizing the number of free parameters) with 2 classical approximations to Bayesian Model Comparison [28]: the Bayesian Information Criterion, which slightly overpenalizes model complexity, and the Akaike Information Criterion, which slightly underpenalizes it. Since these 2 criteria constitute sort of boundaries around the true Bayesian Log Evidence, and since they gave consistent results, we did not include additional criteria.

## Results

### Rat training: 2-day training paradigm

**Fig 2:**
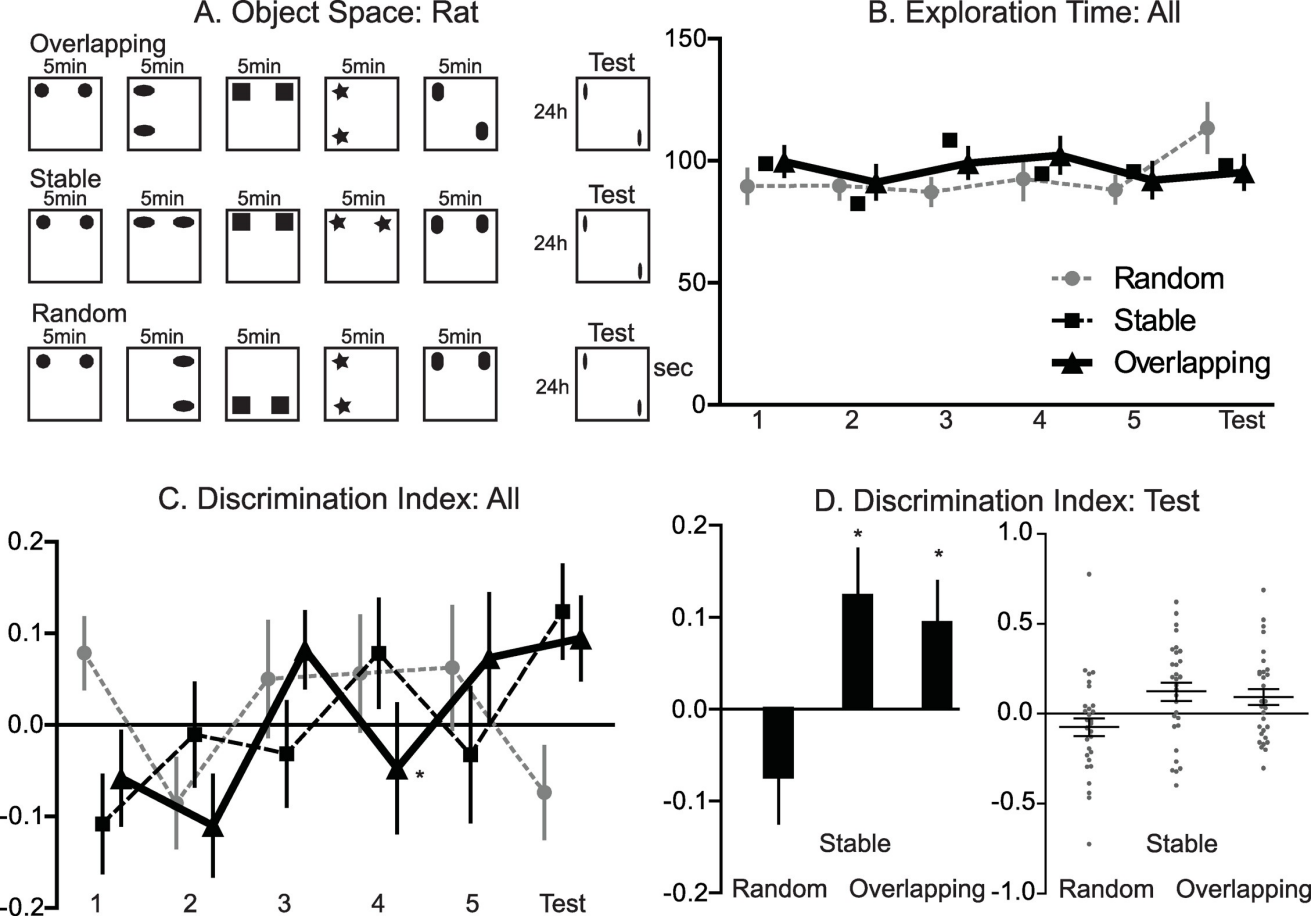
Object space task rat. A. Panel: Trial structures for the 3 different conditions (object identity changed from trial to trial). In the overlapping condition, 1 location remains constant across all sample trials and the test trial, the second location varies. The locations in the last sample trial and in the test trial are equal, thus only cumulative memory across trials will lead to a preference for the less often shown location. In the stable condition, the locations remain the same in all sample trials and 1 object is displaced in the test trial. In the random condition, the locations were pseudo-randomly (controlled for equal appearance of all locations) chosen to not allow extraction spatial patterns. One session consisted of 5 sample trials on 1 day and a test trial 24 hrs later. All locations (i.e., more stable locations in stable and overlapping) were counterbalanced across animals for each condition as well as within an animal across conditions to avoid general place preference effects. B. Panel: Exploration time. The total exploration time remained constant across conditions but a significant effect of trial was observed (*P* = 0.017). C. And D. Panel: DI (for statistical details see main text). The DI across sample and test trials showed a significant condition x trial interaction effect (*P* = 0.011). In the test trial, there was a significant condition effect (*P* = 0.008), and the DI was significantly above chance only for stable and overlapping condition (*P < 0.05). Left and right panel D same data. Data in S1 Data. DI, discrimination index.

Rats were trained for 5 trials in 1 day before being retested 24 hrs later (*n* = 30, Fig 2). There was a small but significant trial effect with increasing total exploration time but no condition effect or conditionXtrial interaction (condition F_2,58_ = 0.27, *P* = 0.76; trial F_5,145_ = 2.87, *P* = 0.017; conditionXtrial F_10,290_ = 1.38, *P* = 0.22; Fig 2B). The DI across all 6 trials showed a significant conditionXtrial interaction but no significant condition effect and only a marginal significant trial effect (condition F_2,58_ = 0.04, *P* = 0.96; trial F_5,145_ = 2.19, *P* = 0.059; conditionXtrial F_10,290_ = 2.35, *P* = 0.011; Fig 2C). Performing the same analysis just on the training data (trial 1–5) exploration time showed condition F_2,58_ = 1.32, *P* = 0.28; trial F_4,116_ = 1.96, *P* = 0.11; conditionXtrial F_8,232_ = 0.83, *P* = 0.58, and DI for condition F_2,58_ = 0.79, *P* = 0.46; trial F_4,116_ = 2.21, *P* = 0.072; conditionXtrial F_8,232_ = 1.57, *P* = 0.13. When focusing on the final test trial, there was a significant condition effect (condition F_2,58_ = 5.29, *P* = 0.008; Fig 2D). In addition, memory performance at the stable condition was significantly above chance (stable: t_29_ = 2.44, *P* = 0.021). DI for the overlapping condition was also significantly above chance (overlapping: t_29_ = 2.09, *P* = 0.045). This was in contrast to random (t_29_ = −1.47, *P* = 0.15). Thus, both overlapping and stable training conditions led to significant memory expression at test 24 hrs later with preferred exploration of the less stable object location, which was not seen in the random condition. For comparison of effect size in DI, we also ran “classic” object displacement with *n* = 8 rats with a 5-min sample period followed 24 hrs later by a test, in this case using the same object for sample and test trial (see Fig 1)

### Mouse training: 5-day training paradigm

**Fig 3:**
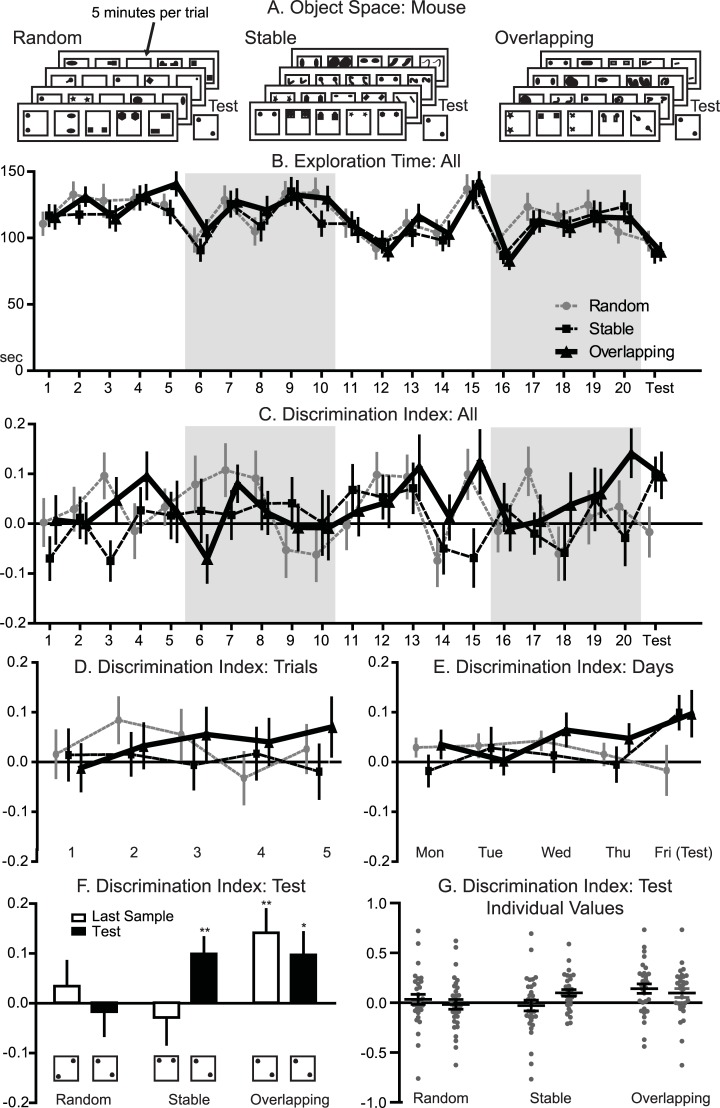
Object space task mouse. A. Panel: Trial structures for the 3 different conditions (object identity changed from trial to trial). In the overlapping condition, 1 location remains constant across all sample trials and the test trial, the second location varies. The locations in the last sample trial and in the test trial are equal. In the stable condition, the locations remain the same in all sample trials and 1 object is displaced in the test trial. In the random condition, the locations were pseudo-randomly chosen (controlled for equal appearance of all locations) to not allow extraction spatial patterns. One session consisted of 5 sample trials for 4 subsequent days and test trial 24 hrs later. All locations (i.e., more stable locations in stable and overlapping) were counterbalanced across animals for each condition as well as within an animal across conditions to avoid general place preference effects. B. Panel: Exploration time over the course of all 20 sample trials and test trial for each condition. Alternating white and grey shaded areas indicate the individual training days and test day. The total exploration time per sample trial remained constant across conditions; however, significant effects of trial, day, and a significant trialXday interaction were observed (condition *P* = 0.59; trial *P* < 0.001; day *P* < 0.001; trialXday *P* < 0.001). C. Panel: DI for all 20 sample trials and test trial across conditions. Alternating white and grey shaded areas indicate individual training days and the test day. D. Panel: DI per sample trial over the course of all 4 training days across conditions. A marginal significant effect for trial has been found (*P* = 0.09). More importantly, a significant conditionXtrial interaction was observed (*P* = 0.042), indicating only a build-up of preference for the less stable location over the daily trials in the overlapping but not stable or random condition. E. Panel: DI for each training day (the 5 sample trials for each training day averaged) and test day per condition. F. Panel: DI at the final training trial and test trial, which showed a significant trialXcondition interaction (*P* = 0.046). Memory performance was significantly above chance level in the overlapping condition for both the last sample trial and test trial (last sample *P* < 0.01; test *P* < 0.05). In the stable condition, only the test trial showed a significant effect (last sample *P* = 0.59; test *P* < 0.01). No significant effects were observed in the random condition (last sample *P* = 0.50; test *P* = 0.73). F and G same data. Data in S2 Data. DI, discrimination index.

Mice were trained with 5-trials a day across 4 days, with a test 24 hrs later (*n* = 31, Fig 3). A 3-trial version was also piloted (*n* = 7, see S2 Fig, S1 Text). No differences in total exploration time were found between conditions or any interaction with condition, but significant trial and day effects were seen during the 20 sample trials (condition F_2,60_ = 0.51, *P* = 0.59; trial F_4,120_ = 15.25, *P* < 0.001; day F_3,90_ = 9.98, *P* < 0.001; conditionXtrial F_8,240_ = 0.42, *P* = 0.85, conditionXday F_6,180_ = 0.35, *P* = 0.85; Fig 3B). In addition, there was a significant trialXday interaction on exploration time (trialXday F_12,360_ = 7.09, *P* < 0.001) but importantly no 3-way interaction (conditionxdayxtrial F_24,720_ = 0.75, *P* = 0.68). In any case, average exploration time remained for each trial comparable with the first day value, showing that, while mice habituate to some degree, they robustly explore the objects throughout the training protocol.

DI across sample trials (20 trials) showed a marginal significant trial effect and more importantly a significant trial x condition interaction (condition F_2,60_ = 0.52, *P* = 0.52; trial F_4,120_ = 2.0, *P* = 0.093; conditionXtrial F_8,240_ = 2.3, *P* = 0.042, Fig 3C), indicating that only in the overlapping condition a build-up was seen during the 5 trials each day. All other main and interaction effects were not significant (day F_3,90_ = 0.93, *P* = 0.43; conditionXday F_6,180_ = 0.87, *P* = 0.52; conditionXdayXtrial F_24,720_ = 1.08, *P* = 0.38 Fig 3D and 3E), except for a significant trialXday interaction (F_12,360_ = 1.97, *P* = 0.026). Concerning the final sample and test trial, there was a significant trialXcondition interaction effect (condition F_2,60_ = 2.0, *P* = 0.14, trial F_1,30_ = 0.12, *P* = 0.73; conditionXtrial F_2,60_ = 0.16, *P* = 0.046; Fig 3F and 3G). One sample t-tests indicated memory performance above chance for the stable condition at test (t_30_ = 3.0, *P* = 0.005). Further, memory performance on both the last sample trial and test in the overlapping condition was significantly above chance, indicating that mice accumulated memory over the course of training, which led to long-term memory expression at 24 hrs (final sample trial t_30_ = 3.0, *P* = 0.005; test t_30_ = 2.16, *P* = 0.039). Finally, no significant effects were observed in the random condition (final sample trial: t_30_ = 0.68, *P* = 0.5; test trial: t_30_ = −0.34, *P* = 0.73). Thus, as in rats, both overlapping and stable training conditions led to significant memory expression at test 24 hrs later with preferred exploration of the less stable object location, which was not seen in the random condition.

### Mouse training: 4-week training paradigm

**Fig 4:**
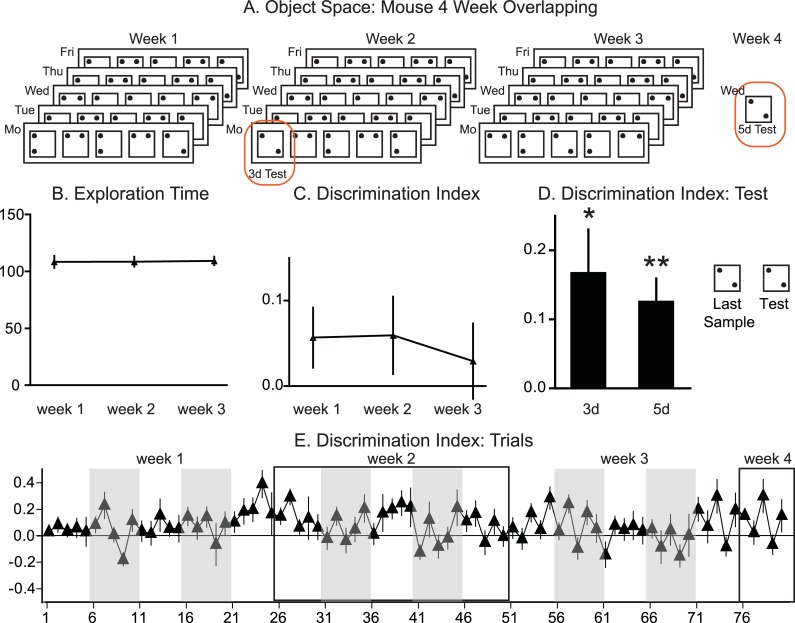
Object space task mouse: 4-week overlapping training. A Panel: Trial structures for the 4-week version of the overlapping condition (object identity changed from trial to trial). Across the 4 weeks, 1 location remains constant across all sample trials and the test trial, the second location varies (5 trials per day, 5 days for the first 3 weeks). The first trial on Monday in week 2 (trial 26) as well as the final trial on Wednesday in week 4 (trial 76) function as 3-day and 5-day test trials, respectively. The stable location was counterbalanced across animals for each condition as well as within an animal across conditions to avoid general place preference effects. B Panel: Exploration time remained stable across the 3 weeks (*P* = 0.96). C Panel: The DI remains stable with preference for the less often shown location across the 3 weeks (*P* = 0.5). D Panel: Test to control for episodic memory effects the locations in the last sample trials and in the tests trial are equal. Both 3 days and 5 days after training, the animals showed a significant cumulative memory effect, with preference for the less often shown location (*P = 0.033, **P = 0.008). E. DI of each trial for the whole 4-week period. Data in S4 Data. DI, discrimination index.

To test if overlapping training led to a memory representation that lasts longer than 24 hrs, 8 mice were additionally trained with 5-trials a day across 25 days, with a final test 5 days later (*n* = 8, Fig 4A). Both the second week’s Monday (trial 26) as well as the final trial (trial 76) were run with the same configuration as the previous sample trial to function as 3-day and 5-day test trials. Exploration time remained stable (week F_2,14_ = 0.4, *P* = 0.96; Fig 4B), and the DI remained positive, indicating decreased preference for the stable location in our overlapping condition (week F_1.2,14_ = 0.5, *P* = 0.53; Fig 4C). The 3-day and 5-day test showed that even after longer periods, the abstracted memory representation is still expressed with both tests above chance level (3-day t_7_ = 2.7, *P* = 0.033; 5-day t_7_ = 3.7, *P* = 0.008; Fig 4D).

### Application of the paradigm to the implanted animals

**Fig 5:**
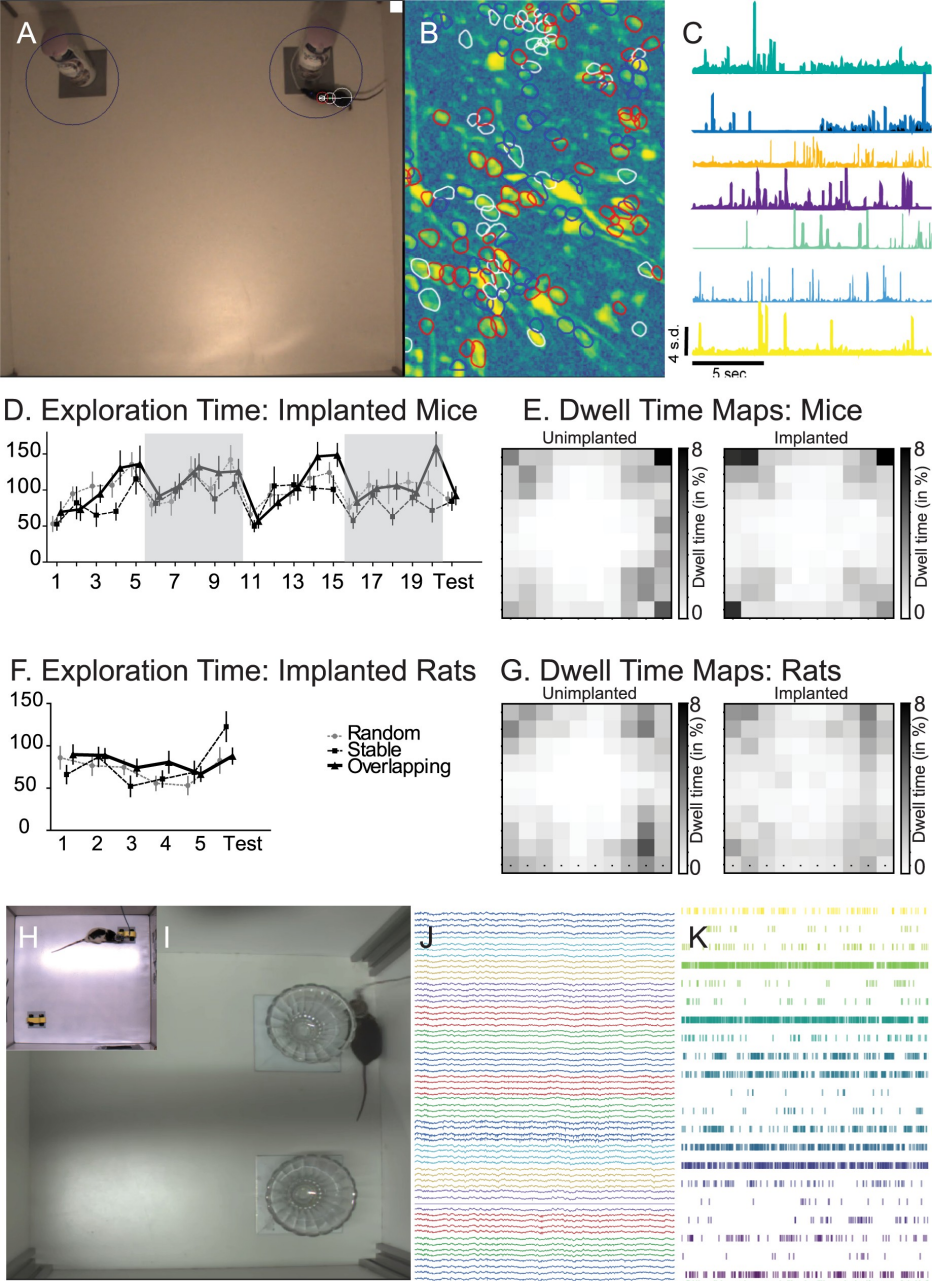
Object space task: Calcium imaging and electrophysiology. Example of a mouse running the object space task with calcium imaging (Inscopix, A), example raw signal (B), and extracted calcium transients (C). D shows exploration times in implanted mice (Data in S6 Data) and E dwell time maps for a random set of 20 trial of unimplanted (left) and implanted (right) mice. F shows exploration times and G dwell times maps for rats (Data in S7 Data; G again random sample of 20 videos). Example of a rat (H) and mouse (I) running the object space task with electrophysiological implants, example raw LFP signal (J) and extracted unit activity (K). Further speed and position analysis of implanted and unimplanted animals: mouse implanted (speed 5.559 cm/s ± 2.323 cm/s, at border: 75.1% of frames, at center: 24.9% of frames. Data in S10 Data), mouse unimplanted (speed 7.497 cm/s ± 4.314cm/s, at border: 75.3% of frames, at center: 24.7% of frames. Data in S11 Data), rat implanted (speed 5.689 cm/s± 1.623 cm/s, at border: 69.1% of frames, at center: 30.9% of frames, Data in S12 Data), and rat unimplanted (speed 7.909 cm/s ± 3.629 cm/s, at border: 79.8% of frames, at center: 20.2% of frames. Data in S13 Data). *n* = 3 for rats, *n* = 6 for mice, random 10 trials were chosen for analysis.

We have tested in this protocol animals that were implanted with either a microendoscope (Fig 5A) or with a microdrive enabling the placement of tetrodes in the brain (Fig 5H and 5I). Both techniques enabled the sampling of large ensembles of neurons in the hippocampus and prefrontal cortex. Fig 5B and 5C shows examples of regions of interest (putative neurons) and calcium transient time courses for the microendoscopy experiments. Fig 5J and 5K show, respectively, raw electrophysiological traces, and multiple single-unit spike trains recorded with a multitetrode microdrive. Importantly, key parameters of animal behavior remained in both cases indistinguishable from those computed for unimplanted animals, in terms of exploration time (Fig 5D and 5F), occupancy of the box, in particular with respect to thigmotaxic behavior (dwelling in the center of the box compared to the edges and corners; Fig 5E and 5G) and running speed (Fig 5, legend). Thus, the task is well suited for the investigation of the link between a gradually built memory trace and the activity of neural ensembles.

### Modeling results

We fit the 4 models *M*_1_−*M*_4_ on the data for the stable, random, and overlapping conditions separately (see Computational methods), and found that model *M*_1_, a simple place–object association learning model with 2 parameters (*α*, which regulates the balance between recent and remote memory, and *β*, which regulates the balance between neophilia and neophobia), gives the most parsimonious account of both rat (Table 1) and mouse (Table 2) behavior in this task.

**Table 1:** Results of model fitting on rat behavioral data. AIC and BIC both give model *M*_1_ as winner. All models fit the data significantly better than chance: pR2 score > 0. The table shows mean ± SEM of parameter values without outliers (|*β*|≥1,000).

Model	α	β	γ	m_0_	LL	AIC	BIC	pR2
**M_1_**	0.69 ± 0.04	6.6 ± 8.0			−184.3	560.7	2168.6	0.26
**M_2_**	0.55 ± 0.04	16.0 ± 17.1	0.36 ± 0.04		−182.4	652.7	2876.4	0.27
**M_3_**	0.59 ± 0.03	5.7 ± 3.5	0.55 ± 0.03	26.6 ± 14.5	−148.9	681.7	3458.1	0.40
**M_4_**	0.60 ± 0.04	6.0 ± 6.4		0.90 ± .004	−180.4	648.9	2868.7	0.28

**Abbreviations:** AIC, Akaike Information Criterion; BIC, Bayesian Information Criterion; LL, log-likelihood; M, model; pR2, pseudo R2

**Table 2:** Results of model fitting on mouse behavioral data. AIC and BIC both give model *M*_1_ as winner. All models fit the data significantly better than chance: pR2 score > 0. The table shows mean ± SEM of parameter values without outliers (|*β*|≥1,000).

Model	α	β	γ	m_0_	LL	AIC	BIC	pR2
**M_1_**	0.66 ± 0.04	11.7 ± 6.0			−212.5	611.1	2259.0	0.08
**M_2_**	0.59 ± 0.04	9.4 ± 5.8	0.34 ± 0.04		−211.0	701.1	2957.4	0.09
**M_3_**	0.59 ± 0.04	−0.8 ± 15.2	0.62 ± 0.04	619 ± 411	−199.5	771.1	3615.9	0.14
**M_4_**	0.39 ± 0.03	−15.2 ± 8.2		0.90 ± 0.004	−209.7	698.4	2952.1	0.09

**Abbreviations:** AIC, Akaike Information Criterion; BIC, Bayesian Information Criterion; LL, log-likelihood; M, model; pR2, pseudo R2

**Fig 6:**
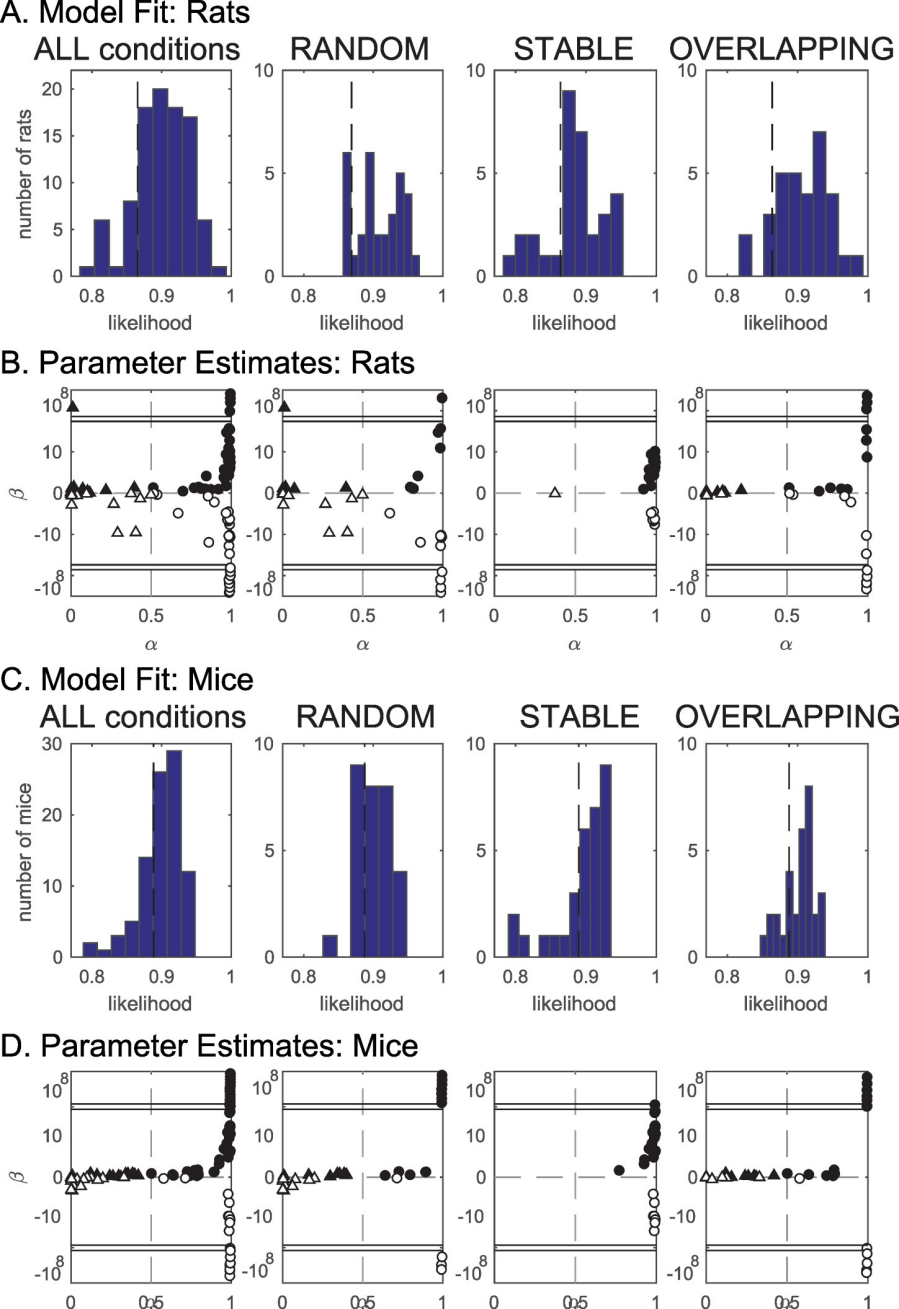
Fit of computational model on data. The optimized model fits the behavior of rats (A–B) and mice (C–D) better than chance. A. Histogram of goodness of fit (model likelihood) for all rats, averaged across conditions (first panel) and in the 3 experimental conditions separately. The vertical dashed line denotes average chance level. Note, however, that for each individual session, the model fits better than that expected by chance for that specific session. B. Optimal values for the model parameters α and β rat experiments. Each symbol corresponds to 1 experiment test (1 animal, 1 condition, conditions may be repeated across animals). For overlapping, the model requires an *α*≪1 in order to reliably accumulate evidence over a large number of trials. In contrast, for stable high values of *α* were obtained. The value of *α* for stable is nevertheless to a large extent immaterial for task performance because recent and remote trials contain the same information (the object configuration does not change). Double horizontal lines indicate y-axis discontinuity (to accommodate for outliers) C. and D. same as A and B for mice. D. Horizontal dashed lines indicate |*β*|≫20 in B and |*β*|≫10 in D. Data in S8 Data.

For all conditions, the model can fit the data better than chance (Table 1, Table 2, Fig 6A and 6C). Interestingly, the distribution of optimized parameters per session shows 3 different tendencies for the 3 different conditions (Fig 6B and 6D):

- For overlapping, the model requires an *α*≪1 in order to reliably accumulate evidence over a large number of trials. Thus, apart from the few outliers, the results show mostly parameters around *β* = 0 and 0<*α*<0.9 (Fig 6B and 6D).
- For stable, the value of *α* is to a large extent immaterial for task performance because recent and remote trials contain the same information (the object configuration does not change). The great majority of individuals have *α*≥0.9 and −10≤*β*≤10 (Fig 6B an d 6D).
- For random, there is no task structure, but the model fit produces parameter distributions not unlike those for overlapping, suggesting that animals can track nonrandom fluctuations in the trial schedule, which is due to counterbalancing and a limited number of trials (Fig 6B and 6D).

**Fig 7:**
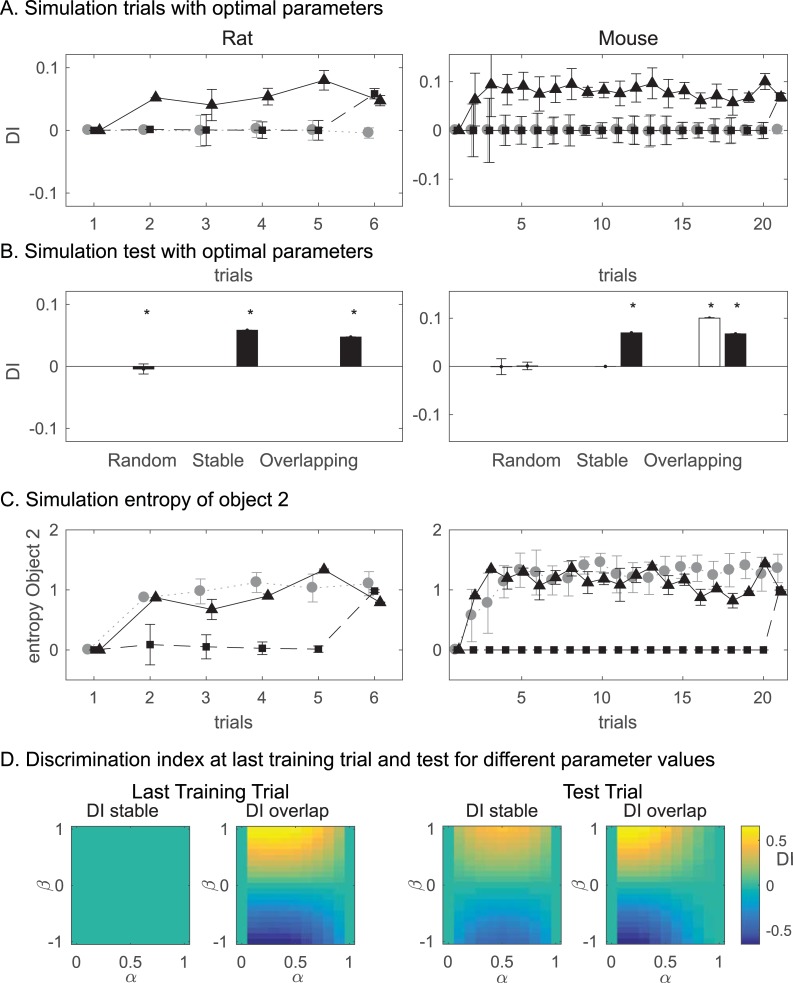
The computational model can reproduce animal performance. The model simulations reproduced the progressive build-up over trials of a significant DI in the overlapping condition for both rats and mice. It also reproduced a positive DI at the test trial for the stable condition. A. DI of model simulated with optimal parameter values (from Tables 1 and 2) for rats and mice. Triangles represent overlapping condition, squares stable, and grey circles random as in Fig 2. B. DI at test trial in model simulations. C. Time course of Entropy of Object 2 in simulations. The model predicts that high DIs are due to a high entropy (i.e., uncertainty) associated to Object 2 (moving object). D. Shows the DI at last training trial and test for different parameter values of α and β. Neophilic behavior (yellow) is obtained for positive β while neophobic behavior (blue) is obtained for negative β. Moreover, α < 0.5 is ideal to obtain a high DI in the overlapping condition, while higher α is acceptable for the test trial of the stable condition. Data in S9 Data. DI, discrimination index.

We then verified that the winning model, model *M*_1_, could reproduce the general behavioral tendencies of the animals when simulated to perform the task on its own, not taking into account the animal’s actual behavior (see Computational methods). We simulated the model on exactly the same series of place–object locations experienced by the animals during different sessions of the task, using the optimized values for parameters *α* and *β*. Fig 7 shows the DI for each condition averaged over all simulated subjects. The model simulations reproduced the progressive build-up over trials of a significant DI in the overlapping condition for both rats and mice. It also reproduced a positive DI at the test trial for the stable condition. The model predicts that this increase is the result of a sudden increase in the entropy (i.e., uncertainty) of the distribution over possible locations in the memory trace associated to object *o*_2_ (Fig 7C), which has been moved only at test trial. Interestingly, the model also predicts that the entropy for object *o*_2_ is also high in the random condition. Nevertheless, the model does not show a high DI in this condition because the entropy for object *o*_1_ is also high, because both objects are constantly moved so that the difference in entropy between the 2 objects is null.

Importantly, with high values of *α* (≥0.9) a build-up of DI is absent in the overlapping conditions (Fig 7D); this is because under these conditions, the model does not rely on remote memory and only focuses on recently memorized information, as if it was driven by episodic memory of the last trial.

We simulated model *M*_1_, with different combinations of parameters. For stable, DI in the test trial becomes highest around *α* = 0.5 (Fig 7D). This value thus represents a sort of optimum in this task, where both recent and remote memories are important information. A higher value of *α* would lead the model to forget even the last training trial and only to rely on the test trial in a way that would be detrimental to performance. Conversely, in stable but very noisy tasks where animals should not update their memory too fast with new information because of the low reliability in this noisy information, the optimum for *α* would be close to 0 [29]. Strikingly, the figure shows that for the overlapping condition, at both last sample and test trials, the highest DI is reached with *α*~0. This illustrates that in the overlapping condition, it is optimal to rely more on remote memory than on recent one.

## Discussion

Knowledge extraction is a gradual process that requires the experience of multiple similar (or overlapping) events; in contrast, episodic memory is by definition based on 1 event [2,5,30,31]. Much research has focused on testing episodic memories [4,5,32] and although some tasks have previously been developed to study cumulative memory, we attempted to develop a task that is simple and easy to implement that allows for a time-saving within-subjects design and makes use of a rodent’s natural behavior without any external motivators. The within-subject design also allows to control for baseline differences in memory capacity or general place preference. We have successfully demonstrated that the object space task can be used to test for cumulative memory and contains both a positive control condition (the stable condition) that can be solved with a single event (as well as cumulative memory) or recency memory as well as a random condition as negative control. By the end of training, both rats and mice show cumulative memory in the overlapping condition, indicated by a positive DI in the test trial. DI in the *stable* condition is, as expected, only biased in the test trial. Finally, object locations in the *random* condition were treated without preference by the animals. And by changing object identity each trial, this task is sure to only test the spatial configurations presented and not any object-based familiarity.

Because the same configuration is used as in the last training trial, the test trial provides a control for any recent memory-like effects in our overlapping condition, clarifying whether the animal uses accumulated memory over the course of learning instead of their most recent experience to guide their behavior. If the animal shows no preference for either object location at the test trial, it can mean 2 things. Either the animal behavior is guided by remembering its most recent experience or the animal has not acquired a long-term, cumulative memory. Even though we cannot assume that the encoding strengths for the stable and overlapping condition are exactly the same, the stable condition does help to differentiate these 2 effects since if the animal can retain a memory of the most recent experience but not a cumulative memory, it still will be above chance in this condition. One could argue that the different training conditions result in different types of memories (e.g., abstracted for overlapping and episodic for stable) and thus a direct comparison with an ANOVA is not warranted, and only a *t* test to chance for each condition is critical to test for significant memory expression. Here, however, this distinction is of no importance since both approaches show significant results. Thus, all 3 conditions together (overlapping, stable and random) enables us to test if an animal under current conditions can remember an event and/or a cumulative memory. We further have expanded the approach in mice and showed that the abstracted memory in the overlapping condition is not only expressed 24 hrs later but is retained for longer time periods, as seen in the 3-day and 5-day tests. At first glance, a DI of approximately 0.1 may seem like a small effect; however, a DI of 0.1 corresponds to animals exploring the moved object 30% more than stable object. Since we change object identity from trial to trail, this task is very different from conventional object-place memory. It tests for subtle difference in spatial statistics instead of the location of an individual object, thus similar effect sizes as in other experiments are not expected. However, in a separate experiment using the conventional paradigm with the same objects and in this case our 5-min sample produces a positive DI at test similar to the values seen in our paradigm (see Fig 1).

The formation of a cumulative memory trace over multiple trials is further supported by fitting a computational model with a parameter α capturing the time scale at which memories are formed or decayed. In the absence of further input, the model would predict that a memory trace of one trial would decay in a time (expressed in number of further trials) $T∼\left(\frac{1}{\alpha }\right)$. Thus, values of *α*~0.1−0.3 (Fig 6) indicate integration of information over multiple trials.

Interestingly, fitting the model for the random condition yields a similar distribution for the values of α, suggesting that animals keep track of object locations in the last few trials and that exploration behavior is affected by fluctuations in the frequency of different locations in the pseudo-random schedule. Thus, while the overlapping condition depends critically on memory accumulation, accumulation is present in all other conditions, including the random one. The use of a pseudo-random schedule (avoiding “runs” of multiple trials with the same object positions) is geared exactly at minimizing such effects. Even a pseudo-random schedule, however, cannot completely compensate for such effects due, for example, to the limited number of possible locations and the limited number of trials. The fact that an overall effect on DI is not observed in that condition and that a simple model as presented here can account for the fluctuations observed in behavior shows that the random condition is a useful control for our main experimental conditions.

While mice require multiple sample trials across multiple days to acquire cumulative memory in this task, rats require just 1 day of training consisting of 5 sample trials in total. Despite this difference in training duration and the definite slower learning curve in mice, we see it as an advantage that this task can be used in both rodent types. Several studies have compared performance in various (complex) tasks in rats and mice and often concluded that mice cannot perform as well as rats [33–35]. However, as we show in our task, by adapting the protocol, mice are able learn this task and retain the information over longer time periods, thereby expanding the opportunities for the use of this task in numerous animal models, taking advantage of the extensive molecular and genetic toolbox currently available for mice. Despite these differences in training duration, we expect that learning in this task underlies similar mechanisms in both rats and mice. However, we cannot draw any conclusions on this until further research has been conducted.

In addition to adapting the task to rats and mice, we developed a software to track the exploration behavior and allow for online scoring of exploration periods. The program automatically reads in predefined trial structures and only informs the experimenter about the objects and locations used right before each trial. Combining this approach with interleaved testing of several animals during 1 experimental session, we effectively blind the experimenter with respect to the condition in the current trial and therefore enable them to score exploration behavior online without introducing an experimenter bias.

In the future, this task will allow for the investigation of the neural circuits contributing to cumulative and event memory. In contrast to water-based paradigm such as that of Richards and colleagues [18], this task is well-suited for electrophysiological recording of brain activity during learning (see Fig 5). Further, this task is especially suitable as memory conditions tapping into memory accumulation versus event memory can be presented in the same spatial layout and with very similar overall behavior, as indicated by the lack of difference in total exploration time across conditions. This will allow the investigation of how the brain represents such statistical patterns ranging from random movement, extractible statistics to very stable representations, expanding on previous research such as [36]. Using stable object configuration across a week, they provided tantalizing evidence for “memory” cells during test trials, in which 1 object was moved. Similar analysis performed within the different object space task conditions could help differentiate how stable an object has to be for such a phenomenon to be observed.

Previous studies have provided evidence that the hippocampus is more involved in the processing of recent experiences that include episodic details, whereas the prefrontal cortex accumulates information from multiple similar experiences, thereby creating a more stable but also more generalized memory over time [1,19,21,37]. We can hypothesize that successful performance on the overlapping condition involves the integration of multiple or all events in the prefrontal cortex, thereby creating a stable representation of the overlapping object location in space. While the classic version of our stable condition, namely 24-hr object-displacement memory, is usually described as a hippocampal-dependent task [38–41], we cannot assume that our stable condition is also dependent on the hippocampus due to the increased number of sample phases. Object-displacement memory requires the animal to experience only 1 event; in the object space task the animal experiences multiple events of the same spatial configuration. Thus, the animal can solve this task by using both its most recent experience and the cumulative memory of the events.

In conclusion, the object space task can be used to study cumulative memory in both rats and mice. Rats require 1 day of training to acquire a cumulative memory while mice require multiple days of training in order to learn this task. Although we can speculate about a critical role of both prefrontal cortex and hippocampus to acquire cumulative memory in the object space task for both rodent types, the neural mechanisms underlying memory performance should be determined next.

## Supporting information

S1 Fig Object space task materials. Examples of objects used in the object space task. Objects vary in size, width, texture, and material. Objects were placed in 2 of the 4 corners. On the right: example of the object scorer program with pop-up pretrial (top) and with scoring (bottom).
(TIF)

S2 Fig Object space mouse 3 sample trials per day. Left panel: Trial structures for the 3 different conditions. In the overlapping condition, 1 location remains constant across all sample trials and the test trial, the second location varies. The locations in the last sample trial and in the test trial are equal. In the stable condition, the locations remain the same in all sample trials and 1 object is displaced in the test trial. In the random condition, the locations were pseudo-randomly chosen to not allow extraction spatial patterns. One session consisted of 3 sample trials for 4 subsequent days and test trial 24 hrs later. Top right panel: Exploration time over the course of all 12 sample trials and test trial per condition. Exploration time was not significantly different between conditions but did show a significant trial effect (trial *P* < 0.001). Mid-right panel: DI averaged across training days and test day for each condition. DI averaged for each day showed a significant effect of condition (condition *P* = 0.014). Bottom right panel: DI at the final sample trial and test for each condition. A significant effect of condition was found (condition *P* = 0.033). Performance in the overlapping condition was above chance at the final sample trial but not at the test (final sample trial: *P* = 0.006; test: *P* = 0.27), Furthermore, we did not observe a significant increase in memory performance on the stable condition test (final sample trial: *P* = 0.57; test: *P* = 0.18, random final sample trial: (*P* = 0.16; test: *P* = 0.99), indicating that there was no 24-hr long-term memory effect after training. Data in S3 Data. DI, discrimination index.
(EPS)

S3 Fig DI at 10-min test. A. Panel: Rat DI at 10-min test. DI was calculated from 10 min of exploration during the test. A marginal significant effect was found for condition (*P* = 0.08) with a significant increase in memory performance in the stable condition (*P* = 0.017). B. Panel: Mouse DI at 10-min test. Focusing on the final sample trial and test, a marginal trial X condition effect was found (*P* = 0.064). In the stable condition, memory performance was significantly increased during test (*P* < 0.01). Performance at the final sample trial was significantly increased in the overlapping condition (*P* = 0.014) and a marginal effect was observed at test (*P* = 0.077). Data in S1 and S2 Data. DI, discrimination index.
(EPS)

S1 Data All rat data in Fig 2. (XLSX)

S2 Data All 5 trial/day mice data in Fig 3. (XLSX)

S3 Data All 3 trial/day mice data in S2 Fig. (XLSX)

S4 Data All mice data 4 week paradigm. (XLSX)

S5 Data All rat data for simple OD. OD, object displacement.
(XLSX)

S6 Data Implanted mouse exploration times in Fig 5. (XLSX)

S7 Data Implanted rat exploration times in Fig 5. (XLSX)

S8 Data All data from Fig 6. (XLSX)

S9 Data All data from Fig 7. (XLSX)

S10 Data Implanted mouse location by time for dwell time maps in Fig 5. (TXT)

S11 Data Unimplanted mouse location by time for dwell time maps in Fig 5. (TXT)

S12 Data Implanted rat location by time for dwell time maps in Fig 5. (TXT)

S13 Data Unimplanted rat location by time for dwell time maps in Fig 5. (TXT)

S1 Text Statistics for 3 trial/day condition in mice and 10-min test for mice and rats. (DOCX)

## Abbreviations

CS: conditioned stimulus
DI: discrimination index
