# Supplementary figures and images for: The object space task shows cumulative memory expression in both mice and rats
Source: PLoS Biol. 2019 Jun 17;17(6):e3000322. doi: 10.1371/journal.pbio.3000322 (PMC6597117; doi:10.1371/journal.pbio.3000322)

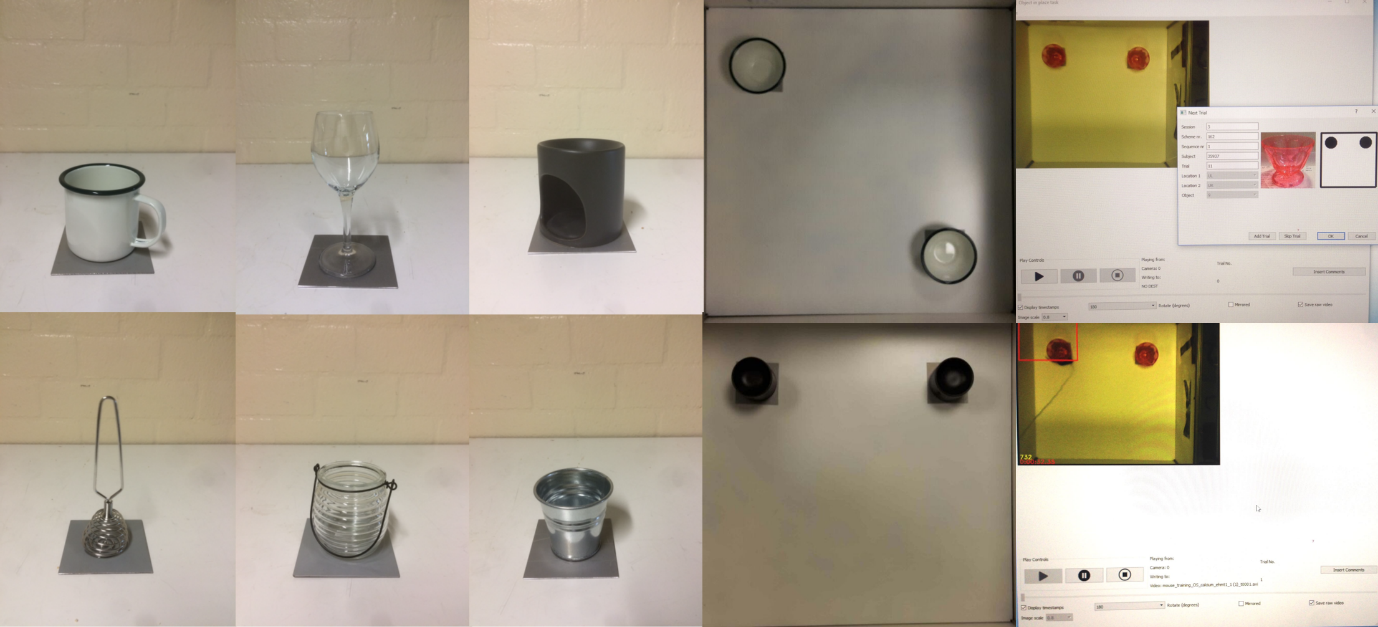

Supplement: S1 Fig — Examples of objects used in the object space task. Objects vary in size, width, texture, and material. Objects were placed in 2 of the 4 corners. On the right: example of the object scorer program with pop-up pretrial (top) and with scoring (bottom). (TIF) [file pbio.3000322.s001.tif]
