# Supplementary material for: The object space task shows cumulative memory expression in both mice and rats
Source: PLoS Biol. 2019 Jun 17;17(6):e3000322. doi: 10.1371/journal.pbio.3000322 (PMC6597117; doi:10.1371/journal.pbio.3000322)
Supplement: S1 Text — (DOCX) [file pbio.3000322.s017.docx]

**The Object Space Task shows cumulative memory expression in both mice and rats**

**Supplemental Materials**

Lisa Genzel#^1,2^, Evelien Schut*^1,2^, Tim Schröder*^1^, Ronny Eichler*^1,2^, Mehdi Khamassi^3^, Angela Gomez^1^, Irene Navarro Lobato^1^, Francesco Battaglia^1^

* equal contribution

# corresponding author: [L.Genzel@donders.ru.nl](mailto:L.Genzel@donders.ru.nl),

1 Donders Institute for Brain Cognition and Behaviour, Radboud University, Nijmegen/Netherlands

2 RadboudUMC, Nijmegen, Netherlands

3 Institute of Intelligent Systems and Robotics, Sorbonne Université, CNRS, Paris, France

**Mouse training: 3 sample trials per day**

*Results:*

Initially, we trained mice (n=7) with 3x 5min sample trials per day for each condition (S2 Fig). Exploration time of animals over the course of training was not significantly different between conditions but did show a significant trial effect (condition F_2,12_=0.8, p=0.47; trial F_12,72_=14.3, p<0.001; conditionXtrial F_24,144_=0.56, p=0.95). Discrimination index averaged for each day showed a significant effect of condition (condition F_2,12_=6.2, p=0.014, day F_4,24_=0.84, p=0.51; conditionXday F_8,48_=0.6, p=0.78). Focusing on the final training and test trial, again a significant effect of condition was found (condition F_2,12_=4.6, p=0.033, trial F_1,6_=1.75, p=0.23; conditionXtrial F_2,12_=1.3, p=0.31). Performance in the *overlapping* condition was above chance at the final sample trial but not at the test trial (final training trial: t_6_=4.2, p=0.006; test trial: t_6_=1.2, p=0.27), Furthermore since we did not observe even a numerical effect on the *stable* condition (final training trial: t_6_=0.6, p=0.57; test trial: t_6_=-1.5, p=0.18, *random* final training trial: t_6_=-1.6, p=0.16; test trial: t_6_=-0.1, p=0.99), indicating that there was no 24hr long-term memory effect after training. Together these results suggest that more extensive training is needed for mouse subjects, therefore we chose to train the mice on 5 sample trials per day instead of 3.

**Rat and mouse training: 10min test**

Animals were allowed to explore for 10 minutes during the test (S3 Fig ). Focusing on the test trial in rats, a marginal significant effect was found for condition (_F2,58_=2.64, p=0.08). Additional analyses showed no effects on the random condition (t_29_=-0.74, p=47). Further, the discrimination index for the stable condition was significantly above chance, indicating a 24hr memory effect, whereas no significant effects were found in the overlapping condition (*stable* t_29_=2.53, p=0.017, *overlapping* t_29_=1.58, p=0.13). However, in the 5min test we did find a significant effect in the overlapping condition (p<0.05). This indicates that rats spend more time exploring the moving object versus the stable object in the first 5 minutes of test, which clearly indicates a memory effect. However, in the last 5 minutes of the test, they have the tendency to return to exploring the stable object location more.

In mice, focusing on the last sample trial and test, a marginal trial X condition interaction effect was found (trail F_1,30_=0.06, p=0.8; condition F_2,60_=1.56 p=0.22; trial X condition F_2,60_=2.88, p=0.064). In addition, memory performance was significantly increased in the stable condition at test, indicating 24hr memory can still be observed with the 10min test (*stable* final sample trial t_30_=0.289 p=0.78; test t_30_=3.01, p<0.01; *random* final sample trial t_30_=0.68, p=0.5; test t_30_=-0.53, p=0.59). Performance at the final sample trial was significantly increased in the overlapping condition (t_30_=2.62, p=0.014) and a marginal effect was observed at test (t_30_=1.83,p=0.077). Thus, even though we can still observe the memory effects with a 10min test compared to a 5in test, the observed effects are stronger for the first 5 minutes compared to the whole 10min test.

Compared to the data from the 5min test, these results from both rats and mice indicate that analyses from the full 10 minutes of test are not able to demonstrate the memory effects as strongly as we observe during the first 5 minutes of the test. Both rats and mice spend more time exploring the object location that is more novel to them in the beginning of the test. Then the time spent exploring the familiar object increases as the 10min test progresses. Hence, a 5min test is a better representation of memory performance in the object space task.
